# Treatment expectations but not preference affect outcome in a trial of CBT and exercise for pain

**DOI:** 10.1080/24740527.2017.1384297

**Published:** 2017-10-11

**Authors:** Marcus John Beasley, Elizabeth Alice Ferguson-Jones, Gary John Macfarlane

**Affiliations:** ^a^ Epidemiology Group, School of Medicine, Medical Sciences and Nutrition, University of Aberdeen, Aberdeen, United Kingdom; ^b^ Aberdeen Centre for Arthritis and Musculoskeletal Health, University of Aberdeen, Aberdeen, United Kingdom

**Keywords:** Placebo response, treatment preference, expectation, randomized controlled trials, treatment effect, nonspecific effects

## Abstract

**Background**: Patients’ beliefs and attitudes toward a treatment can affect treatment response. In unblinded trials this can affect outcomes.

**Aims**: The aim of this analysis was to examine the association between treatment preference and expectation and outcome in a trial of pain treatments.

**Methods**: In a randomized trial (ISRCTN67013851) of four treatments for chronic widespread pain, participants were asked which they would prefer and what improvement they expect from each. The proportion of participants reporting positive health outcomes at three time points after treatment were compared between those matched or unmatched with their preference and between those with and without expectation for improvement. Odds ratios were calculated adjusted for baseline characteristics associated with preference and expectation.

**Results**: Four hundred forty-two participants were recruited to the trial (69.5% female). The proportion reporting positive outcomes among participants matched to their preference compared to those unmatched was 33.3% vs. 34.4% at the end of treatment (adjusted odds ratio [aOR] = 0.80, 95% confidence interval [CI], 0.44–1.46), 34.4% vs. 29.0% at 3 months (aOR = 1.23, 95% CI, 0.67–2.26), and 34.8% vs. 30.3% at 2 years (aOR = 1.31, 95% CI, 0.70–2.46). The proportion of participants reporting positive outcomes among those expecting improvement compared to those not expecting improvement was 36.6% vs. 15.0% at the end of treatment (aOR = 2.03, 95% CI, 1.07–3.85), 34.1% vs. 13.2% at 3 months (aOR = 2.31, 95% CI, 1.22–4.38), and 32.8% vs. 19.1% at 2 years (aOR = 1.16, 95% CI, 0.67–2.36).

**Conclusions**: Treatment preference had no clear effect on outcomes, but expectation did. These results could inform future approaches to management, and researchers assessing treatments should take into account this expectation effect.

## RÉSUMÉ

**Contexte**: Les croyances et les attitudes des patients à l’égard d’un traitement peuvent influencer la réponse à ce traitement. Dans des essais sans insu, il peut y avoir un effet sur les résultats.

**Buts**: Le but de cette analyse était d’étudier le lien entre les préférences et les attentes à l’égard du traitement, et le résultat obtenu dans un essai portant sur les traitements de la douleur.

**Méthodes**: Dans un essai randomisé (ISRCTN67013851) portant sur quatre traitements pour la douleur chronique généralisée, on a demandé aux participants quelle était leur préférence quant au traitement, ainsi que l’amélioration qu’ils attendaient de chacun de ces traitements. Une comparaison de la proportion de participants ayant rapporté des résultats positifs sur leur santé à trois moments différents après le traitement a été effectuée entre ceux qui ont reçu le traitement qu’ils préféraient et ceux qui ont reçu un traitement autre que celui qu’ils préféraient, ainsi qu’entre ceux qui s’attendaient à une amélioration et ceux qui n’avaient pas de telles attentes. Les rapports de cotes ont été calculés et ajustés selon les caractéristiques de départ en ce qui concerne la préférence et les attentes.

**Résultats**: Le nombre de participants recrutés pour cet essai était de 442 (69,5 % de femmes). La proportion de participants ayant rapporté un résultat positif parmi ceux qui ont reçu le traitement qu’ils préféraient comparativement à ceux qui ont reçu un traitement autre que celui qu’ils préféraient était de 33,3 % comparativement à 34,4 % à la fin du traitement (RC ajusté 0,80, 95 % IC 0,44-1,46); de 34,4 % comparativement à 29,0 % après trois mois (RCa 1,23, 0,67 – 2,26) et de 34,8 % comparativement à 30,3 % après deux ans (RCa 1,31, 0,70 – 2,46). La proportion de participants ayant rapporté des résultats positifs parmi ceux qui s’attendaient à une amélioration comparativement à ceux qui n’avaient pas de telles attentes était de 36,6 % comparativement à 15,0 % à la fin du traitement (RCa 2,03, 1,07-3,85), de 34,1 % comparativement à 13,2 % après trois mois (RCa 2,31, 1,22*-4,38), et de 32,8 % comparativement à 19,1 % après deux ans (RCa 1,16, 0,67-2,36).*

**Conclusions**: La préférence en matière de traitement n’a pas eu d’effet clair sur les résultats, contrairement aux attentes. Ces résultats pourraient inspirer les approches futures en matière de prise en charge, tandis que les chercheurs qui évaluent des traitements devraient tenir compte de l’effet des attentes.

## Introduction

When a patient receiving treatment for pain gets better, he or she might have done so through specific hypothesized mechanisms arising from the treatment. However, there are other reasons why a treatment might appear to work. The natural history of the condition may be such that symptoms resolve without treatment. Regression to the mean happens when we select people at a time when they are at an extreme on some measurement and only measure outcomes in those people. Some components of a treatment are not specific to that treatment but instead are common to many, such as attention from a medical practitioner,^1^ so effects due to these components cannot be uniquely ascribed to the treatment. Finally, there is a collection of effects that are due to the beliefs a patient has about the treatment. These can include the expectancy that the treatment will have an effect and preference for one treatment over another. As well as direct effects that beliefs and attitudes have on outcomes, these factors may induce demand characteristics in self-reported answers to questions such as, “Do you feel better?” which might bias responses.

When we look at all these together—natural history, regression, common factors, treatment beliefs, demand characteristics—it could be said that we are looking at the placebo response, a response that is not due to the effect of treatment.^2^ Only once these components of the placebo response have been accounted for can we say whether we have discovered a specific treatment effect or not. This is one reason for conducting randomized trials comparing outcomes between people receiving a treatment and those not receiving the treatment. In such trials, participants are randomly allocated to either a group receiving the treatment of interest or a control group not receiving that treatment (though they may be receiving an active treatment for comparison or a treatment that is thought to be inert). People in the control group may get better for reasons other than the treatment, but any response in the treatment group over and above that in the control group can be attributed to the treatment. However, this only applies when the two groups do not differ with respect to all factors thought to comprise the placebo effect, including beliefs and expectations the participant has about the treatment, as well as artefacts such as regression to the mean. In unblinded trials, participants know whether they are in a control group or whether they are receiving a treatment. In an unblinded trial comparing two or more active treatments, participants may have differing expectations for the effectiveness of the treatments and may have a preference for one of them. Preference for a particular treatment has been noted as a potential threat to the validity of unblinded trials,^3^ and the effects of expectation on pain have been well documented.^4,5^ One example of the effect of expectation in an experimental setting is analgesia induced by the use of an inert cream with healthy subjects who believe it to be an effective treatment for pain.^6^

Prior to allocation to a treatment group in a randomized trial, information can be collected from participants about which treatment they would prefer and also about the outcome they would expect from each of the treatments. Then it is possible to see whether outcomes differ according to whether or not participants received their preferred treatment and whether or not they had high expectations for the treatment they received. The MUSICIAN Study is one such randomized trial that has previously reported effective treatments for pain^7,8^ and that collected information on preference and expectations. The aim of the current analysis was to see whether patient-reported improvement in health (the main outcome) in a randomized trial for cognitive–behavioral therapy (CBT), exercise, or both treatments for patients with chronic widespread pain (CWP) was associated with nonspecific factors of treatment, namely, whether participants received the treatment they preferred and whether they expected improvement for the treatment they received.

## Materials and methods

The MUSICIAN Study was a 2 × 2 randomised trial of CBT or exercise for people with CWP. The full details and results have been reported previously.^7–9^ The trial compared three active treatment arms—a CBT program delivered by telephone (tCBT), an exercise program, or both exercise and tCBT delivered simultaneously versus treatment as usual (TAU). The tCBT consisted of an initial assessment by telephone with a therapist, followed by seven weekly sessions and follow-up sessions at 3 and 6 months. The exercise program was conducted at a leisure facility gym and was led by a fitness instructor with whom participants could meet once a month.^8^ A screening survey of patients registered at selected doctors’ practices in two areas of the UK, with approximately 15 000 respondents, was used to identify and recruit 442 participants. Eligible participants reported CWP (according to the definition in the American College of Rheumatology criteria for fibromyalgia)^10^ for which they had consulted their general practitioner. Participants were at least 25 years old, and exclusion criteria included contraindications to exercise and psychiatric disorder.^8^ Confirmation of eligibility for the study and treatment allocation was conducted at clinic visits with a research nurse. The study received full ethical approval from the Cheshire Research Ethics Committee (reference number: 07/Q1506/61), the trial registration number was ISRCTN67013851, and all participants gave informed consent.

### Measures

Participants were randomly allocated to one of the three active treatments (tCBT, exercise, or both combined) or TAU. Immediately before treatment allocation, participants were asked, “If you had to choose the treatment(s) to have which would you choose?” Participants could select one answer from a list of five: cognitive–behavioral therapy alone, exercise therapy alone, both treatments, no preference, or don’t know. Those who were subsequently allocated to their preferred treatment were classed as matched, and those who were allocated to a treatment other than their stated preference were classed as unmatched. Because the aim of this analysis was to examine the effects of receiving a preferred treatment, those with no preference were also allocated to the unmatched group because they could not be said to have received their preferred treatment if they did not have one. For two additional sensitivity analyses, participants who did not have a treatment preference were first removed from the unmatched group and secondly included in the matched group.

Participants were also asked four questions before allocation: “If you receive cognitive–behavioral therapy only, how do you think this will affect your pain after 6 months of treatment?” with “exercise therapy only,” “both exercise and cognitive–behavioral therapy,” or “neither therapy (i.e., receive the usual care given by your general practitioner)” substituted for “cognitive–behavioral therapy only.” Participants could select one answer from a list of five for each question: “It would be much improved,” “It would be a little improved,” “It would be neither better nor worse,” “It would be a little worse,” or “It would be much worse.” Participants were classed as having had an expectation of improved if the response was, “It would be much improved” or “It would be a little improved” for the treatment to which they were subsequently allocated and classed as no change or worse otherwise.

The following baseline characteristics were also collected prior to treatment allocation: age, gender, Chronic Pain Grade (CPG),^11^ passive and active coping measured by the Vanderbilt Pain Management Inventory,^12^ the Chalder Fatigue Scale,^13^ psychological distress (measured by the General Health Questionnaire [GHQ]-12),^14^ the Sleep Problem Scale,^15^ and the Tampa Scale for Kinesiophobia.^16^ Where single item responses were missing on any of these scales, scores for that item were imputed as the mean score of the other items for that measure.

The main outcome measure of the trial was patient-reported improvement in global health, on a seven-point scale ranging from *very much better* through *no change* to *very much worse*. The main outcome was collected at three time points: at the end of treatment and 3 months and 2 years after the end of treatment. Responses were dichotomized with *very much better* or *much better* classed as a positive outcome.

### Statistical methods

First, associations of treatment preference and expectation with baseline characteristics were identified in order to use as adjusting factors when looking at their associations with outcomes. Differences in gender and CPG by treatment preference were tested with chi-square tests. The other baseline characteristics were treated as continuous and differences by preference were tested with analysis of variance. Where a difference was found, Tukey’s honestly significant difference was used to identify which preferences differed, and the difference in scores are reported with 95% confidence intervals (CIs). Differences in proportions of genders and CPG with expectations of improvement or no change or worse for each treatment were tested with chi-square tests. We used *t* tests to test for differences in baseline characteristics for those expecting improvement or no change or worse for each treatment, and where differences were found they are reported with 95% CIs.

The proportion of participants reporting a positive outcome (much better or very much better) among those matched to their treatment preference was compared to those unmatched among all those allocated to active treatment at all time points and then for each treatment group separately. Participants allocated to TAU were not included in this analysis, because the option was not given in the question eliciting treatment preference. Logistic regression was used to calculate odds ratios with 95% CIs of a positive outcome in those matched compared to those unmatched. Odds ratios were then adjusted for baseline factors associated with treatment preference. Two sensitivity analyses were conducted, firstly removing those without any treatment preference from the unmatched group and, secondly, including those without a preference in the matched group.

The proportion of participants with a positive outcome among those with an expectation of improvement for the treatment they received was compared to those without among all participants at all time points and then for each treatment group separately. Logistic regression was also used to calculate odds ratios with 95% CIs of positive outcome in those with expectations of improvement for the treatment to which they were allocated, compared to those without. Odds ratios were then adjusted for baseline characteristics associated with expectations of improvement for a particular treatment.

## Results

### Treatment preference

Combined CBT and exercise was the most preferred of the active treatments, with 199 participants (45.0%) choosing this treatment prior to allocation (Table 1). CBT alone was the least preferred treatment, with only 20 participants (4.5%) choosing this treatment. Participants choosing different treatments differed on a number of baseline characteristics. Exercise alone was the preferred treatment for males, with 59 (43.7%) choosing this, whereas combined CBT and exercise was the preferred treatment for females, with 152 (49.5%) choosing this option. Those who preferred CBT alone had higher scores than those who preferred exercise alone for psychological distress (4.7 vs. 2.3, difference 2.3, 95% CI, 0.8–3.8), passive coping (34.0 vs. 27.6, difference 6.4, 95% CI, 3.0–9.7), sleep problems (11.6 vs. 8.1, difference 3.5, 95% CI, 1.0–6.1), fatigue (22.4 vs. 18.4, difference 4.0, 95% CI, 1.6–6.5), and kinesiophobia (38.8 vs. 35.1, difference 3.7, 95% CI, 1.0–6.3). Those preferring CBT alone also had higher scores than those preferring combined CBT and exercise for passive coping (34.0 vs. 30.0, difference 4.0, 95% CI, 0.7–7.2) and kinesiophobia (38.8 vs. 36.0, difference 2.7, 95% CI, 0.3–5.2).

**Table 1.** Characteristics by treatment preference.

		Preferred treatment				
		CBT	Exercise	Both treatments	No preference	*P* value
*n* (%)		20 (4.5)	144 (32.6)	199 (45.0)	79 (17.9)	
Age, mean (SD)		52.9 (14.0)	57.4 (13.1)	55.4 (13.1)	57.1 (12.7)	ns
Gender	Males (%)	6 (4.4)	59 (43.7)	47 (34.8)	23 (17.0)	<0.01
	Females (%)	14 (4.6)	85 (27.7)	152 (49.5)	56 (18.2)	
CPG	Grades 1 or 2 (%)	10 (3.8)	94 (35.3)	120 (45.1)	42 (15.8)	ns
	Grades 3 or 4 (%)	10 (5.7)	50 (28.4)	79 (44.8)	37 (21.0)	
GHQ 12, mean (SD)		4.7 (4.2)	2.3 (3.0)	3.7 (3.7)	3.3 (3.7)	<0.01
Passive coping, mean (SD)		34.0 (5.9)	27.6 (7.2)	30.0 (7.2)	30.4 (7.8)	<0.01
Active coping, mean (SD)		23.6 (3.7)	24.9 (4.6)	25.1 (4.1)	24.6 (4.0)	ns
Sleep problems, mean (SD)		11.6 (5.8)	8.1 (5.3)	9.4 (5.7)	10.1 (5.6)	<0.01
Fatigue, mean (SD)		22.4 (7.5)	18.4 (4.9)	20.2 (5.9)	20.4 (6.6)	<0.01
Kinesiophobia, mean (SD)		38.8 (8.6)	35.1 (5.1)	36.0 (4.8)	35.7 (5.7)	0.03

CBT = cognitive–behavioral therapy; ns = not significant; CPG = Chronic Pain Grade; GHQ = General Health Questionnaire.

There was no clear association between being matched to a preferred treatment group and achieving a positive outcome (Tables 2a–2c). The proportion of participants with a positive outcome at all time points was similar among those who were matched to their preferred treatment and those who were unmatched: 33.3% vs. 34.4% at the end of treatment (adjusted odds ratio [aOR] = 0.80, 95% CI, 0.44–1.46), 34.4% vs. 29.0% at 3 months after the end of treatment (aOR = 1.23, 95% CI, 0.67–2.26), and 34.8% vs. 30.3% at 2 years (aOR = 1.31, 95% CI, 0.70–2.46).

**Table 2a.** Outcomes at end of treatment by being matched to preferred treatment.

	Preference	Positive outcome	Negative outcome	OR	Adjusted OR^a^	Adjusted for treatment OR^b^
All active treatments (*n* = 273)	Matched	31 (33.3)	62 (66.7)	0.95 (0.56–1.62)	0.93 (0.54–1.62)	0.80 (0.44–1.46)
	Unmatched	62 (34.4)	118 (65.6)	1 (Ref)	1 (Ref)	1 (Ref)
tCBT (*n* = 87)	Matched	2 (33.3)	4 (66.7)	1.19 (0.20–6.92)	1.13 (0.15–8.30)	
	Unmatched	24 (29.6)	57 (70.4)	1 (Ref)	1 (Ref)	
Exercise (*n* = 92)	Matched	12 (33.3)	24 (66.7)	0.90 (0.37–2.18)	0.65 (0.23–1.86)	
	Unmatched	20 (35.7)	36 (64.3)	1 (Ref)	1 (Ref)	
Combined (*n* = 94)	Matched	17 (33.3)	34 (66.7)	0.69 (0.30–1.61)	0.69 (0.28–1.72)	
	Unmatched	18 (41.9)	25 (58.1)	1 (Ref)	1 (Ref)	

^a^Adjusted for gender, GHQ scores, passive coping, sleep problems, Chalder fatigue scores, and kinesiophobia.

^b^Additionally adjusted for treatment group.

OR = odds ratio; tCBT, telephone-delivered cognitive–behavioral therapy; GHQ = General Health Questionnaire.

**Table 2b.** Outcomes at 3 months by being matched to preferred treatment.

	Expectation	Positive outcome	Negative outcome	OR	Adjusted OR^a^	Adjusted for treatment OR^b^
All active treatments (*n* = 289)	Matched	33 (34.4)	63 (65.6)	1.28 (0.76–2.16)	1.17 (0.67–2.03)	1.23 (0.67–2.26)
	Unmatched	56 (29.0)	137 (71.0)	1 (Ref)	1 (Ref)	
tCBT (*n* = 89)	Matched	4 (66.7)	2 (33.3)	4.64 (0.80–27.00)	7.73 (0.98–60.67)	
	Unmatched	25 (30.1)	58 (69.9)	1 (Ref)	1 (Ref)	
Exercise (*n* = 99)	Matched	10 (26.3)	28 (73.7)	1.20 (0.47–3.06)	1.28 (0.43–3.84)	
	Unmatched	14 (23.0)	47 (77.1)	1 (Ref)	1 (Ref)	
Combined (*n* = 101)	Matched	19 (36.5)	33 (63.5)	1.08 (0.48–2.45)	0.96 (0.39–2.37)	
	Unmatched	17 (34.7)	32 (65.3)	1 (Ref)	1 (Ref)	

^a^Adjusted for gender, GHQ scores, passive coping, sleep problems, Chalder fatigue scores, and kinesiophobia.

^b^Additionally adjusted for treatment group.

OR = odds ratio; tCBT, telephone-delivered cognitive–behavioral therapy; GHQ = General Health Questionnaire.

**Table 2c.** Outcomes at 2 years by being matched to preferred treatment.

	Preference	Positive outcome	Negative outcome	OR	Adjusted OR^a^	Adjusted for treatment OR^b^
All active treatments (*n* = 267)	Matched	31 (34.8)	58 (65.2)	1.23 (0.71–2.11)	1.12 (0.63–1.99)	1.31 (0.70–2.46)
	Unmatched	54 (30.3)	124 (69.7)	1 (Ref)	1 (Ref)	
tCBT (*n* = 82)	Matched	1 (16.7)	5 (83.3)	0.34 (0.04–3.09)	0.29 (0.03–3.08)	
	Unmatched	28 (36.8)	48 (63.2)	1 (Ref)	1 (Ref)	
Exercise (*n* = 92)	Matched	13 (37.1)	22 (62.9)	1.81 (0.73–4.52)	1.54 (0.53–4.49)	
	Unmatched	14 (24.6)	43 (75.4)	1 (Ref)	1 (Ref)	
Combined (*n* = 93)	Matched	17 (35.4)	31 (64.6)	1.51 (0.62–3.66)	1.12 (0.39–3.18)	
	Unmatched	12 (26.7)	33 (73.3)	1 (Ref)	1 (Ref)	

^a^Adjusted for gender, GHQ scores, passive coping, sleep problems, Chalder fatigue scores, and kinesiophobia.

^b^Additionally adjusted for treatment group.

OR = odds ratio; tCBT, telephone-delivered cognitive–behavioral therapy; GHQ = General Health Questionnaire.

The sensitivity analyses similarly showed no clear association between being matched to a preferred treatment group and outcomes. The proportions reporting a positive outcome among those matched to their preferred treatment compared to those not, excluding those with no treatment preference, was 33.3% vs. 35.9% at the end of treatment (aOR = 0.83, 95% CI, 0.43–1.63), 34.4% vs. 31.9% at 3 months after the end of treatment (aOR = 1.16, 95% CI, 0.59–2.26), and 34.8% vs. 30.5% at 2 years (aOR = 1.14, 95% CI, 0.57–2.30). The proportion reporting a positive outcome among those matched compared to those not, including those with no preference in the matched group, was 32.4% vs. 35.9% at the end of treatment (aOR = 0.72, 95% CI, 0.41–1.26), 29.8% vs. 31.9% at 3 months after the end of treatment (aOR = 0.79, 95% CI, 0.45–1.39), and 33.1% vs. 30.5% at 2 years (aOR = 1.16, 95% CI, 0.64–2.10).

### Expectation

Combined CBT and exercise had the highest expectation of improvement, with 355 participants (80.3%) expecting an improvement in pain after 6 months of this treatment (Table 3). Usual care had the lowest expectation of improvement, with 41 participants (9.3%) expecting to be improved. Those expecting improvement from CBT had higher psychological distress than those not expecting improvement (3.8 vs. 2.6, difference 1.1, 95% CI, 0.5–1.8). A greater proportion of those with low CPG expected improvement from exercise compared to those with a high CPG (81.6% vs. 72.7%). Those expecting improvement from exercise compared to those not expecting improvement had lower passive coping (28.8 vs. 31.6, difference −2.8, 95% CI, −4.5 to −1.2) and lower kinesiophobia (35.1 vs. 38.2, difference −3.1, 95% CI, −4.3 to −1.9). Females were more likely to expect improvement from combined CBT and exercise treatment than males (83.1% vs. 74.1%). Those with low CPG were more likely to expect improvement from combined treatment than those with high CPG (83.5% vs. 75.6%). Those expecting improvement from combined treatment also had lower levels of passive coping than those not expecting improvement (29.0 vs. 31.2, difference −2.2, 95% CI, −3.9 to −0.48) and kinesiophobia (35.2 vs. 38.0, difference −2.9, 95% CI, −4.1 to −1.6).

**Table 3.** Characteristics of those with expectations of improvement for each treatment.

			Expectation		
			No change or worse	Improved	*P* value
CBT	Number of participants (%)		215 (48.6)	227 (51.4)	
	Age, mean (SD)		56.7 (13.1)	55.8 (13.0)	ns
	Gender	Males (%)	75 (55.6)	60 (44.4)	ns
		Females (%)	140 (45.6)	167 (54.4)	
	CPG	1 or 2 (%)	134 (50.4)	132 (49.6)	ns
		3 or 4 (%)	81 (46.0)	95 (54.0)	
	GHQ, mean (SD)		2.6 (3.3)	3.8 (3.7)	<0.01
	Passive coping, mean (SD)		36.2 (5.1)	35.4 (5.5)	ns
	Active coping, mean (SD)		28.8 (7.7)	30.1 (7.1)	ns
	Sleep problems, mean (SD)		9.0 (5.8)	9.4 (5.4)	ns
	Fatigue, mean (SD)		19.3 (5.5)	20.2 (6.2)	ns
	Kinesiophobia, mean (SD)		36.2 (5.1)	35.4 (5.5)	ns
Exercise	Number of participants (%)		97 (21.9)	345 (78.1)	
	Age, mean (SD)		57.6 (13.1)	55.9 (13.0)	ns
	Gender	Male (%)	31 (23.0)	104 (77.0)	ns
		Female (%)	66 (21.5)	241 (78.5)	
	CPG	1 or 2 (%)	49 (18.4)	217 (81.6)	0.03
		3 or 4 (%)	48 (27.3)	128 (72.7)	
	GHQ, mean (SD)		3.3 (3.7)	3.2 (3.5)	ns
	Passive coping, mean (SD)		31.6 (8.3)	28.8 (7.0)	<0.01
	Active coping, mean (SD)		24.4 (4.5)	25.0 (4.1)	ns
	Sleep problems, mean (SD)		10.0 (6.1)	9.0 (5.4)	ns
	Fatigue, mean (SD)		20.5 (5.8)	19.5 (5.9)	ns
	Kinesiophobia, mean (SD)		38.2 (5.8)	35.1 (5.0)	<0.01
Both treatments	Number of participants (%)		87 (19.7)	355 (80.3)	
	Age, mean (SD)		57.8	55.9	ns
	Gender	Male (%)	35 (25.9)	100 (74.1)	0.03
		Female (%)	52 (16.9)	255 (83.1)	
	CPG	1 or 2 (%)	44 (16.5)	222 (83.5)	0.04
		3 or 4 (%)	43 (24.4)	133 (75.6)	
	GHQ 12, mean (SD)		3.2 (3.7)	3.2 (3.5)	ns
	Passive coping, mean (SD)		31.2 (8.9)	29.0 (6.9)	0.01
	Active coping, mean (SD)		24.4 (4.8)	25.0 (4.1)	ns
	Sleep problems, mean (SD)		9.6 (6.3)	9.1 (5.4)	ns
	Fatigue, mean (SD)		20.1 (5.7)	19.7 (5.9)	ns
	Kinesiophobia, mean (SD)		38.0 (5.9)	35.2 (5.0)	<0.01
Usual care	Number of participants (%)		401 (90.7)	41 (9.3)	
	Age, mean (SD)		56.3 (13.2)	55.4 (11.8)	ns
	Gender	Male (%)	118 (87.4)	17 (12.6)	ns
		Female (%)	283 (92.2)	24 (7.8)	
	CPG	1 or 2 (%)	245 (92.1)	21 (7.9)	ns
		3 or 4 (%)	156 (88.6)	20 (11.4)	
	GHQ 12, mean (SD)		3.2 (3.5)	2.9 (3.7)	ns
	Passive coping, mean (SD)		29.3 (7.3)	30.5 (7.9)	ns
	Active coping, mean (SD)		24.9 (4.2)	24.3 (4.7)	ns
	Sleep problems, mean (SD)		9.2 (5.5)	9.1 (6.5)	ns
	Fatigue, mean (SD)		19.9 (5.8)	18.8 (6.4)	ns
	Kinesiophobia, mean (SD)		35.8 (5.2)	35.6 (6.7)	ns

CBT = cognitive–behavioral therapy; ns = not significant; CPG = Chronic Pain Grade; GHQ = General Health Questionnaire.

There was an increased odds of a positive outcome among participants who had an expectation of improvement for the treatment to which they were allocated compared to those not (Tables 4a–4c) at the end of treatment (36.6% vs. 15.0%, aOR = 2.03, 95% CI, 1.07–3.85) and 3 months later (34.1% vs. 13.2%, aOR = 2.31, 95% CI, 1.22–4.38). For those receiving CBT, there was increased odds of reporting a positive outcome in those with an expectation of improvement compared to those not at the end of treatment (40.4% vs. 17.5%, aOR = 2.95, 95% CI, 1.05–8.30) and 3 months later (47.8% vs. 16.3%, aOR = 4.52, 95% CI, 1.65–12.39). For those receiving exercise alone, there was an increased odds of reporting a positive outcome in those with an expectation of improvement compared to those not 3 months after the end of treatment (29.3% vs. 8.3%, aOR = 4.52, 95% CI, 1.09–27.2).

**Table 4a.** Outcomes at end of treatment by treatment expectation.

	Expectation	Positive outcome	Negative outcome	OR	Adjusted OR	Adjusted for treatment OR
All (*n* = 360)	Improved	78 (36.6)	135 (63.4)	3.28 (1.93–5.59)	3.41^a^ (1.97–5.89)	2.03^b^ (1.07–3.85)
	No change or worse	22 (15.0)	125 (85.0)	1 (Ref)	1 (Ref)	1 (Ref)
tCBT (*n* = 87)	Improved	19 (40.4)	28 (59.6)	3.20 (1.17–8.72)	2.95^c^ (1.05–8.30)	
	No change or worse	7 (17.5)	33 (82.5)	1 (Ref)	1 (Ref)	
Exercise (*n* = 92)	Improved	26 (37.1)	44 (62.9)	1.58 (0.55–4.53)	1.80^d^ (0.57–5.74)	
	No change or worse	6 (27.3)	16 (72.7)	1 (Ref)	1 (Ref)	
Combined (*n* = 94)	Improved	32 (38.1)	52 (61.9)	1.44 (0.35–5.95)	1.66^e^ (0.34–8.08)	
	No change or worse	3 (30.0)	7 (70.0)	1 (Ref)	1 (Ref)	
TAU (*n* = 87)	Improved	1 (8.3)	11 (91.7)	1.04 (0.11–9.54)	—	
	No change or worse	6 (8.0)	69 (92.0)	1 (Ref)	—	

^a^Adjusted for gender, GHQ scores, CPG, passive coping, and kinesiophobia.

^b^Adjusted for gender, GHQ scores, CPG, passive coping, kinesiophobia, and treatment group.

^c^Adjusted for GHQ scores.

^d^Adjusted for CPG, passive coping, and kinesiophobia.

^e^Adjusted for gender, CPG, passive coping, and kinesiophobia.

OR = odds ratio; tCBT = telephone-delivered cognitive–behavioral therapy; TAU = treatment as usual; GHQ = General Health Questionnaire; CPG = Chronic Pain Grade.

**Table 4b.** Outcomes at 3 months by treatment expectation.

	Expectation	Positive outcome	Negative outcome	OR	Adjusted OR	Adjusted for treatment OR
All (*n* = 387)	Improved	75 (34.1)	145 (65.9)	3.41 (2.01–5.78)	3.35^a^ (1.95–5.76)	2.31^b^ (1.22–4.38)
	No change or worse	22 (13.2)	145 (86.8)	1 (Ref)	1 (Ref)	1 (Ref)
tCBT (*n* = 89)	Improved	22 (47.8)	24 (52.2)	4.71 (1.74–12.75)	4.52^c^ (1.65–12.39)	
	No change or worse	7 (16.3)	36 (83.7)	1 (Ref)	1 (Ref)	
Exercise (*n* = 99)	Improved	22 (29.3)	53 (70.7)	4.57 (0.99–21.10)	5.43^d^ (1.09–27.2)	
	No change or worse	2 (8.3)	22 (91.7)	1 (Ref)	1 (Ref)	
Combined (*n* = 101)	Improved	31 (35.6)	56 (64.4)	1.00 (0.31–3.24)	0.67^e^ (0.18–2.48)	
	No change or worse	5 (35.7)	9 (64.3)	1 (Ref)	1 (Ref)	
TAU (*n* = 98)	Improved	0 (0.0)	12 (100.0)	0.61 (0.00–4.38)*	—	
	No change or worse	8 (9.3)	78 (90.7)	1 (Ref)	—	

^a^Adjusted for gender, GHQ scores, CPG, passive coping, and kinesiophobia.

^b^Adjusted for gender, GHQ scores, CPG, passive coping, kinesiophobia, and treatment group.

^c^Adjusted for GHQ scores.

^d^Adjusted for CPG, passive coping, and kinesiophobia.

^e^Adjusted for gender, CPG, passive coping, and kinesiophobia.

OR = odds ratio; tCBT = telephone-delivered cognitive–behavioral therapy; TAU = treatment as usual; GHQ = General Health Questionnaire; CPG = Chronic Pain Grade.

**Table 4c.** Outcomes at 2 years by treatment expectation.

	Expectation	Positive outcome	Negative outcome	OR	Adjusted OR	Adjusted for treatment OR
All (*n* = 361)	Improved	67 (32.8)	137 (67.2)	2.07 (1.26–3.39)	1.85^a^ (1.11–3.07)	1.26^b^ (0.67–2.36)
	No change or worse	30 (19.1)	127 (80.9)	1 (Ref)	1 (Ref)	1 (Ref)
tCBT (*n* = 82)	Improved	17 (40.5)	25 (59.5)	1.59 (0.64–3.96)	1.40^c^ (0.54–3.59)	
	No change or worse	12 (30.0)	28 (70.0)	1 (Ref)	1 (Ref)	
Exercise (*n* = 92)	Improved	23 (32.4)	48 (67.6)	2.04 (0.62–6.74)	1.64^d^ (0.47–5.75)	
	No change or worse	4 (19.1)	17 (81.0)	1 (Ref)	1 (Ref)	
Combined (*n* = 93)	Improved	25 (30.9)	56 (69.1)	0.89 (0.25–3.24)	0.64^e^ (0.15–2.67)	
	No change or worse	4 (33.3)	8 (66.7)	1 (Ref)	1 (Ref)	
TAU (*n* = 94)	Improved	2 (20.0)	8 (80.0)	1.85 (0.34–9.97)	—	
	No change or worse	10 (11.9)	74 (88.1)	1 (Ref)	—	

^a^Adjusted for gender, GHQ scores, CPG, passive coping, and kinesiophobia.

^b^Adjusted for gender, GHQ scores, CPG, passive coping, kinesiophobia, and treatment group.

^c^Adjusted for GHQ scores.

^d^Adjusted for CPG, passive coping, and kinesiophobia.

^e^Adjusted for gender, CPG, passive coping, and kinesiophobia.

OR = odds ratio; tCBT = telephone-delivered cognitive–behavioral therapy; TAU = treatment as usual; GHQ = General Health Questionnaire; CPG = Chronic Pain Grade.

## Discussion

This secondary analysis of the results from a trial of treatments for patients with CWP found that participants who were randomly allocated to their preferred treatment did no better than those who were not. It also found that participants who had a prior expectation for improvement from the treatment group to which they were allocated did do better than those who did not expect improvement. This was particularly so for those allocated to CBT treatment, and particularly at the end of treatment and 3 months after ending treatment. The effect remained after adjusting for baseline characteristics associated with expectations that might have had an effect on outcomes. Generally, trial participants had a greater preference for exercise or for combined exercise and CBT than for CBT alone. They also had greater expectation of improvement for these treatments compared to CBT or usual care from a general practitioner.

A strength of this study is that participants were asked to indicate their preferences and expectations prior to allocation to treatment, so their choices were not influenced by the treatment they were eventually assigned. Likewise, randomization ensured that participants had no choice over which treatment they received. The trial, however, was not designed to measure the effects of patient attitudes toward treatment. There may have been small effects of preference or expectation that the trial was not powered to detect. In particular, there were only a small number of participants who had a preference for CBT, so though there was no overall effect of receiving a preferred treatment, there may have been a benefit for those who preferred CBT and received it. In addition, usual care was not given as an option for participants to choose as a preference, which means it was not possible to measure any preference effect among those receiving it. This, along with the small number of participants choosing some treatments, meant that interaction effects of preference and expectation were not examined in this analysis. And, of course, we did not randomly assign treatment preferences and expectations to the participants. Another weakness of the study is that preference was measured by a single question, to which participants could only make one response—some participants may have had equal preference for more than one treatment option. The preference question also did not measure strength of preference for or against different treatment options. It is possible that preference effects might be only be seen in those with strong preferences. It should also be noted that the question used to elicit prior expectations of treatment effectiveness asked about expectations for pain rather than overall health, which was the main outcome of the study.

Despite these limitations, the findings of this study were largely in agreement with previous studies looking at participants’ preferences in the context of randomized controlled trials for musculoskeletal conditions. In trials of exercise,^17^ acupuncture,^18^ and physical and behavioral therapies^19^ for back pain, outcomes were not associated with receiving preferred treatment. However, George and Robinson^19^ did find that those with no strong treatment preference had better outcomes for pain and disability than those with a preference. Additionally, Foster et al.^20^ found no effect of patients’ treatment preferences in a trial of acupuncture or exercise for knee osteoarthritis, and Stewart et al.^21^ concluded there was no evidence that preferences moderated the effect of exercise for chronic whiplash. These previous studies were looking at conditions other than CWP and at treatments other than exercise or CBT, and the questions used to elicit preference would have been different depending on the design of the trial. The current study adds to these by showing that the lack of a preference effect in pain trials is robust for different patient groups and different methods of measuring preference.

The current study also adds to a large body of evidence that suggests that expectations affect outcomes in randomized trials of pain treatments. In one study, expectations for a cognitive–behavioral intervention were found to be associated with outcomes in patients with fibromyalgia or chronic low back pain.^22^ In trials looking at both physical and behavioral therapies for low back pain, prior expectations for improvement have been found to be associated with effectiveness of the treatment^19^ and disability and satisfaction.^23^ Expectations have also been shown to be associated with outcomes in pain trials of acupuncture,^18,24,25^ massage,^24^ and paracetamol.^26^ These studies investigated the effects of expectation on a range of outcomes for different treatments and conditions using different methods for measuring expectations, with the Credibility/Expectancy Questionnaire being a commonly used measure.^27^ What the current study adds to the existing evidence is some suggestion that the effect of expectations might differ over time and that they may be greater for some treatments than for others.

There are separate implications for clinical practice and for design of research studies from these results. Whereas in research we may seek to minimize and rule out effects due to nonspecific factors such as preference and expectations to determine the specific mechanisms that make a treatment work, in clinical practice we may seek to maximize these effects to promote better outcomes. Although we found that preference did not have an effect on outcomes, it should not be assumed that preference is not an important factor when choosing treatment for patients with pain conditions. The context of a participant in a randomized trial is quite different from that of a patient visiting a clinician, and we cannot be sure that preference would not have an effect on outcome in the clinic. In addition, there are other considerations when deciding on a treatment other than measurable health outcomes, and it may still be beneficial to treat patients according to preference. However, when improvement in overall health outcomes is a consideration, this does show that patients will do better when receiving a treatment where they expect to do better. So clinicians should consider giving pain patients treatments for which they have higher expectations. Future treatments for pain that target expectations for pain should also be explored.^28^

For designers of research studies who are seeking to determine treatment effects, preference and expectation can be considered nuisance factors that should be controlled or adjusted for. The results of this study imply that, though preference may not have much of an effect on outcomes, differing expectations for different treatment arms need to be taken into account. At the very least, expectation for the effectiveness of treatment a participant receives in a trial should be measured and reported when discussing results to aid in their interpretation.^29^ New research designs can be used to assess how much of an observed effect is due to expectation and how much is due to specific effects, such as two-stage designs where participants are randomized to receive either a randomly chosen treatment or a treatment for which they have higher expectations.^3^ And in randomized trials where an active placebo control group is used as a comparator, they should be chosen to match the treatment group for expectations of effectiveness.^30^

There are a number of questions left unanswered about expectation and its effects on outcomes. A first question would be how best to measure it. Another is what outcomes can be affected by expectation. We also need to be clear about how it works and whether it can be explained by reporting bias. Some previous research has shown that adherence to treatment is not a mediator between expectation and outcomes,^26,31^ so other mediators need to identified. One difficulty lies in not being able to assign participants randomly to high or low expectations, so other research designs need to be used. In this study, we found a number of factors associated with positive or negative expectations for treatment, in particular, higher psychological distress among those expecting improvement from CBT and lower levels of passive coping and kinesiophobia among those expecting improvement from exercise. Future research could examine how expectations for treatment are related to personality factors, previous experience of treatment, and comorbidities. Another question is whether expectations can change over the course of treatment and then whether those changed expectations can affect outcomes or whether changes proceed from rather than precede changes in outcome. Finally, further research into treatments that are specifically designed to modify patients’ expectations of the effectiveness of treatment should be explored.^28^

In conclusion, the results of this study demonstrate that though patients’ preferences for different treatments has no clear effect on health outcomes, beliefs about the effectiveness of the treatment they receive do. This is particularly so for those receiving CBT. These results could inform targeting of treatments for patients with pain and when assessing treatments, researchers should always take into account this expectation effect.
